# Challenges of Single-Reference Benchmarks for Clinical Reasoning in Large Language Model–Based Decision Support

**DOI:** 10.2196/99019

**Published:** 2026-11-13

**Authors:** Jiawei Zhou, Hanqing He, Weijie Yu, Zihan Zhong, Qiang Ju, Shuhang Wang, Xiaochuan Wang, Ning Li

**Affiliations:** 1 Cancer Hospital of Chinese Academy of Medical Sciences, Panjiayuan Nanli 17, Beijing, 100021, China, 86 0316-591-8787; 2 Baichuan Inc, Beijing, China

**Keywords:** clinical decision, large language model, benchmark, oncology, reasoning

## Abstract

This viewpoint addresses developers and evaluators of large language model (LLM)–based clinical decision support systems. Although recent benchmarks increasingly evaluate multiple clinical reasoning domains, many still rely substantially on reference decision agreement—whether model outputs conform to predefined decisions. Our aim is to clarify when such agreement remains useful, when it becomes insufficient for complex or preference-sensitive decisions, and how it can be complemented by reasoning-centered assessment. Clinical decision-making often depends on structured reasoning under uncertainty, integrating probabilistic evidence, contextual constraints, and patient values. Using oncology as an illustrative domain, we argue that some decision variability is a structural feature of clinical reasoning rather than noise or error. Used alone in such settings, single-reference benchmarks may conflate reasonable divergence with incorrectness and reward answer imitation over transparent reasoning. We conclude that reference decision agreement should be retained for tasks with stable ground truth but supplemented by evidence grounding, reasoning quality, patient-centeredness, and safety checks for open-ended clinical decision support. This multidimensional approach is intended to support safer and more clinically meaningful evaluation.

## Introduction

The evaluation of large language model (LLM)–based medical AI has entered a new stage of methodological consolidation. A growing number of benchmarks now attempt to assess model performance on complex clinical decision-making tasks, often using expert-curated cases and structured scoring schemes. Recent work has moved beyond simple multiple-choice accuracy toward longitudinal and multidimensional assessment of differential diagnosis, testing, management, and other reasoning domains [1,2]. These efforts represent important progress toward clinically meaningful evaluation.

However, the validity of an evaluation framework does not primarily depend on the surface realism of its inputs. An additional unresolved question is how clinical decision-making should be benchmarked when several decisions may be defensible. Despite recent progress, many benchmarks retain a central organizing principle: whether a model’s final recommendation matches a predefined reference answer. Model performance is then quantified by agreement, accuracy, or consensus with this reference, often aggregated across cases into summary scores.

This approach embeds an assumption that, for a given clinical scenario, one decision can serve as the reference against which alternatives are judged. That assumption is reasonable for tasks dominated by factual recall, guideline recognition, staging, or narrow diagnostic classification, but it can become insufficient when the target is complex, uncertain, or preference-sensitive clinical reasoning. In these settings, performance also depends on the ability to identify missing information, weigh competing risks, justify decisions in context, and recognize the limits of available evidence. Disagreement does not always indicate error, nor does agreement necessarily demonstrate high-quality clinical reasoning.

We argue that reference decision agreement is insufficient when used alone to evaluate open-ended clinical reasoning in LLM-based decision support systems [1,3,4]. This viewpoint is directed to benchmark developers, clinical AI researchers, clinicians, and regulators; it does not address narrow classification systems or rule-based clinical decision support systems with stable reference standards. Our aims are to delimit the tasks for which single-reference scoring remains valid, explain why it becomes insufficient in high-uncertainty or preference-sensitive decisions, and propose an operational reasoning-centered supplement. Using oncology as an example, we illustrate that nonunique decision-making can be a structural feature of medicine. Treating every clinical decision as a problem with one correct answer may penalize reasonable variability and obscure the reasoning processes that determine decision quality in real-world care.

## When Single-Reference Evaluation Becomes Insufficient

Gong et al [5] identified 39 medical AI benchmarks published between 2017 and 2025. We use this set as the basis for Table S1 in Multimedia Appendix 1 and examine a different question: how these benchmarks define acceptable clinical outputs. For each benchmark, we reviewed the primary report and recorded its reference output structure, whether the evaluation permits multiple acceptable responses, and whether it explicitly assesses reasoning, uncertainty, patient preferences, safety, and guideline concordance. Many of these benchmarks nevertheless continue to score model outputs against a predefined answer key or reference response. This approach is well suited to settings in which ground truth is stable and unambiguous. However, when applied to open-ended clinical decision-making, it may impose determinism where several decisions remain clinically defensible. A single preferred output can collapse a multidimensional reasoning process and implicitly treat deviation from the reference decision as failure.

In real clinical settings, such deviation often reflects structured differences in clinical reasoning rather than error: information prioritization, interpretation of uncertainty, tolerance for risk, or weighting of patient preferences. By design, single-reference scoring tables are blind to these factors. They cannot distinguish between a poorly reasoned answer that happens to align with the reference and a well-reasoned alternative that does not.

Importantly, reference-based evaluation remains appropriate for tasks such as factual recall, guideline recognition, staging, and narrowly scoped diagnostic classification, where the solution space is well defined and correctness is relatively unambiguous. Its validity is therefore task-dependent. For closed-form tasks with stable standards, decision agreement can remain primary; for open-ended management tasks characterized by uncertainty, competing options, or preference sensitivity, it should be combined with reasoning, evidence grounding, safety, and patient-centered metrics.

As a result, when used alone in open-ended management tasks, reference decision agreement becomes a blunt instrument: it may conflate clinically reasonable variability with incorrectness and reduce clinical judgment to answer matching. Some recent benchmarks operationalize reasoning by decomposing reference answers into predefined steps and scoring step coverage [6]. This is useful when the steps are clinically necessary, but it can still replace answer matching with step matching if alternative valid pathways are not allowed. The remaining question is whether gains reflect stronger medical reasoning, better conformity to a reference pathway, or both.

The distinction parallels machine learning research on outcome versus process supervision [7]. The medical setting adds a specific challenge: clinically acceptable reasoning may follow more than one pathway, and a fluent rationale may still be post hoc or unsupported. Recent work on reasoning fidelity and expert-aligned rationale assessment reinforces the need to evaluate evidence-conclusion coherence rather than fluency alone [8,9].

## Clinical Decision-Making Is Not Always Deterministic

Contemporary medicine is grounded in scientific evidence, but many clinical decisions remain probabilistic and value sensitive. Stable reference standards exist for some tasks, whereas open-ended management decisions often require context-sensitive reasoning that integrates incomplete information, probabilistic evidence, and patient values (Figure 1).

This epistemic structure directly challenges evaluation frameworks that assume a single correct decision per case. Clinical reasoning is therefore inherently individualized. Decisions are shaped not only by biomedical data but also by patient preferences, comorbidities, functional status, and social context. Moreover, clinical decision-making is not an isolated cognitive act. It often involves shared deliberation between physicians and patients, with the relative weight of physician expertise and patient values varying across clinical settings and health care systems. Under such conditions, the notion of a single objectively correct medical decision is difficult to sustain.

**Figure 1.**
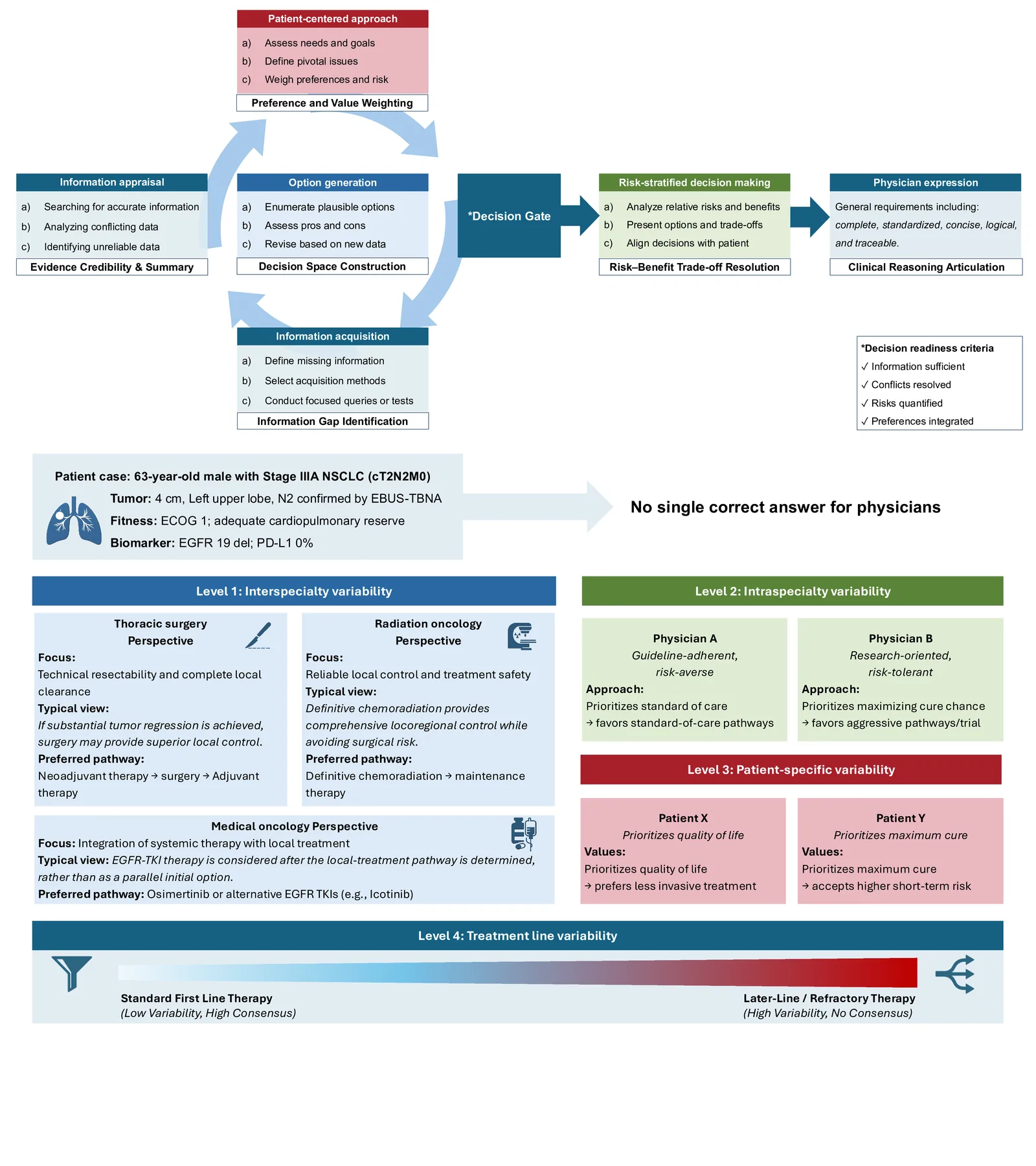
Clinical reasoning framework and an example illustrating nonunique clinical decision-making. The upper panel outlines a patient-centered clinical reasoning process, highlighting iterative evidence appraisal, option generation, risk-benefit evaluation, and integration of patient preferences rather than a single correct answer. The lower panel shows a stage IIIA non–small cell lung cancer (NSCLC) case as an example, demonstrating that clinically reasonable decisions may differ across specialties, physicians, patients, and treatment lines. Colors denote corresponding sources of variability, emphasizing that clinical decision-making is inherently nonunique. EBUS-TBNA: endobronchial ultrasound–guided transbronchial needle aspiration; ECOG: Eastern Cooperative Oncology Group; EGFR: epidermal growth factor receptor; PD-L1: programmed death ligand 1; TKI: tyrosine kinase inhibitor.

As a direct consequence of this nondeterministic reasoning structure, variability in medical decisions should be understood as a structural feature of clinical practice rather than as noise or error. Different clinicians may reasonably arrive at different decisions when evaluating the same patient, based on differences in experience, risk tolerance, and value judgments. Likewise, even the same clinician may make different decisions for patients who appear similar according to guideline-defined criteria.

This phenomenon persists even in oncology, one of the most evidence-rich domains of modern medicine. In a duplicate review of identical cases from the Targeted Agent and Profiling Utilization Registry (TAPUR) Study, recommendations were fully or partially concordant in 79% of cases and discordant in 21%; disagreements frequently concerned the strength of treatment evidence [10]. In a prospective Japanese quality improvement study, 12 molecular tumor boards independently assessed the same 50 simulated cases; mean concordance with centrally developed consensus recommendations was 62% and was substantially lower for genomic alterations supported by lower levels of evidence [11]. These findings do not imply that every divergent recommendation is equally valid. Rather, they show that decision variability becomes more likely when the available evidence is limited or open to interpretation and that population-level evidence must still be interpreted for individual patients whose characteristics and priorities cannot be fully captured by categorical definitions.

Figure 1 further illustrates how such variability may arise within a fully specified case: a 63-year-old man with biopsy-confirmed lung adenocarcinoma, cT2bN2M0 stage IIIA disease, single-station nonbulky N2 involvement confirmed by endobronchial ultrasound–guided transbronchial needle aspiration, Eastern Cooperative Oncology Group performance status 1, adequate cardiopulmonary reserve, an EGFR (epidermal growth factor receptor) exon 19 deletion, and programmed death ligand 1 expression of 0%. The case is assumed to be potentially resectable after multidisciplinary review. Within that shared context, clinicians may still differ in how they weigh a neoadjuvant strategy followed by surgery against definitive concurrent chemoradiotherapy, while remaining explicit about the evidence, resectability assumptions, toxicity, and patient goals.

Beyond physician-level factors, patient-specific considerations further shape decision-making. Differences in tolerance for toxicity, preferences regarding quality of life, and attitudes toward uncertainty may shift the balance between aggressive and conservative approaches, even when guideline eligibility criteria are identical. Finally, as the disease course evolves, treatment line–dependent reassessment may alter the perceived risk-benefit trade-off, resulting in changes in consensus over time for the same patient.

The set of defensible options also changes with treatment setting and should not be collapsed into one decision context. After complete resection of EGFR-mutated stage IB-IIIA disease, adjuvant osimertinib has demonstrated an overall survival benefit [12]. For unresectable stage III disease without progression after chemoradiotherapy, consolidation osimertinib has demonstrated a major progression-free survival benefit [13]. In advanced disease, both osimertinib with or without platinum-pemetrexed and aumolertinib have phase 3 evidence, whereas afatinib and gefitinib remain earlier-generation alternatives with different efficacy and central nervous system activity [14,15]. Aumolertinib improved progression-free survival compared with gefitinib, but no direct trial establishes superiority over afatinib [15]. Access and toxicity may influence selection, but they do not make these options evidence equivalent.

Together, these cases demonstrate how multiple clinically acceptable decisions can emerge from a shared clinical reasoning framework. Divergence arises not from error or nonadherence to evidence but from structured differences in information prioritization, option framing, and value weighting inherent to individualized clinical care. Treating such variability as noise in single-reference evaluation therefore represents a categorical mismatch between how decisions are made and how they are assessed.

## Why Reference Decision Agreement Is Insufficient as a Stand-Alone Metric

Here, reference decision agreement means concordance with a benchmark answer; it is distinct from patient outcomes. Patient outcomes remain crucial downstream criteria, but they are multifaceted—including survival, symptoms, toxicity, function, and quality of life—and may be delayed or confounded by subsequent care. When evaluation frameworks treat open-ended decisions as single-answer problems, a well-reasoned alternative may be penalized while an unsupported answer that matches the reference may be rewarded. Used alone, this can incentivize conformity rather than transparent handling of uncertainty and trade-offs.

Accuracy remains necessary, but it is insufficient in these settings. Agreement with a reference decision does not establish that the supporting clinical rationale is evidence grounded, and disagreement does not by itself establish failure. Single-reference benchmarks can therefore reward reproduction of an expected answer without testing whether a model can identify information gaps, appraise evidence, articulate trade-offs, and adapt to patient values. These reasoning components should be assessed as a complement to decision agreement and patient outcomes, not as their replacement [8,16].

## A Reasoning-Centered and Agent-Compatible Framework for LLM-Based Clinical Decision Support

As Figure 1 illustrates, clinical decision-making is best understood as a reasoning framework that more faithfully reflects how medicine is practiced. Rather than converging on a single “correct” answer, clinical decision-making proceeds through iterative cycles of evidence appraisal, option generation, and risk-benefit trade-off resolution, with explicit integration of patient preferences. Within this framework, decision readiness is achieved when information is sufficient, conflicts are addressed, uncertainty is acknowledged, and values are incorporated—not when a particular option is selected. As a result, different reasoning pathways may reasonably lead to different clinical decisions.

This reasoning-centered perspective is a complementary extension of evaluation: it adds the quality, transparency, and coherence of the clinician-visible reasoning artifact to reference decision and patient outcome measures. Variability is not automatically treated as error, but neither is every divergent answer accepted; the reasoning must remain evidence grounded, safe, and appropriate to the specified context.

Importantly, this framework also provides a natural foundation for agent-based medical AI systems, which are increasingly proposed as a way to move beyond single-turn answer generation. Rather than producing a one-shot recommendation, a reasoning-oriented agent can be evaluated on how it decomposes complex clinical problems into intermediate reasoning steps—such as identifying information gaps, querying additional evidence, generating and revising candidate management options, and explicitly weighing trade-offs under uncertainty. Each of these steps corresponds to established components of clinical reasoning and can be assessed independently of the final decision selected.

From an evaluation perspective, such agents enable more granular assessment. Evaluators may examine whether an agent identifies missing or conflicting information, generates a clinically plausible option set, and justifies its recommendation using accepted principles of risk-benefit assessment and patient-centered care. These properties can be evaluated alongside reference decisions rather than solely through a single preferred outcome.

A preliminary rubric can score six domains from 0 to 2 (Table 1): (1) information sufficiency and identification of gaps, (2) evidence grounding and source quality, (3) construction of a clinically plausible decision space, (4) risk-benefit assessment and uncertainty calibration, (5) integration of patient preferences, and (6) safety and guideline boundaries. A score of 0 indicates absent or unsafe reasoning, 1 indicates partial treatment, and 2 indicates explicit and clinically defensible treatment. The 0 to 12 total should be interpreted only after a safety floor is passed; an unsafe recommendation or fabricated evidence fails the safety gate regardless of the aggregate score.

**Table 1.** Preliminary rubric for assessing clinical reasoning quality^a^.

Domain	0 points	1 point	2 points
Information sufficiency	Relevant information gaps are not identified	Some gaps are identified, but their implications are incompletely addressed	Key information gaps and their implications for decision-making are explicitly identified
Evidence grounding	Recommendations are unsupported or inconsistent with available evidence	Relevant evidence is cited but incompletely appraised or applied	Evidence quality, applicability, and limitations are explicitly considered
Decision space construction	Only 1 option is presented without justification	Alternatives are mentioned but not systematically compared	Clinically reasonable alternatives are identified and compared
Risk-benefit and uncertainty	Risks, benefits, or uncertainty are omitted	Some risks, benefits, or uncertainties are discussed	Major risks, benefits, and uncertainties are explicitly balanced
Patient preferences	Patient preferences are not considered	Preferences are acknowledged but not integrated into the recommendation	Preferences and goals are explicitly incorporated into the decision
Safety and guideline boundaries	The response overlooks important safety or guideline constraints	Relevant constraints are mentioned but incompletely applied	Safety requirements and guideline boundaries are explicitly checked and incorporated

a Each domain is scored from 0 to 2, yielding a total score of 0-12. The total is interpreted only after the safety floor is passed; an unsafe recommendation or fabricated evidence fails the safety gate regardless of the aggregate score.

At least 2 qualified clinicians should score cases independently after calibration with anchor examples; disagreements should be adjudicated, and interrater agreement should be reported. Because a generated rationale is an observable artifact rather than proof of the model’s hidden internal computation, rubric scoring should be complemented by source verification, counterfactual or perturbation testing, and reference decision or patient outcome constraints [8,9].

For the Figure 1 case, a high-scoring response would recognize the need for pathologic and invasive nodal confirmation, preserve both resectable and unresectable pathways until multidisciplinary assessment is complete, distinguish evidence strength across treatment settings, state the relevant safety considerations, and elicit the patient’s priorities. A response that simply lists several EGFR tyrosine kinase inhibitors as equivalent would fail evidence grounding criteria even if one listed option matched the reference answer.

## Conclusions

Clinical decision-making is not always a single-answer problem, yet many medical AI benchmarks retain single-reference scoring. Using reference decision agreement alone can conflate clinically reasonable variability with error and reward answer imitation over transparent handling of uncertainty. In open-ended contexts, evaluation should assess both the clinical acceptability of the decision and the quality of the supporting reasoning, including evidence appraisal, risk-benefit assessment, uncertainty, patient values, and safety boundaries. Extending evaluation beyond answers is therefore a practical requirement for developing LLM-based decision support systems that can be meaningfully assessed before real-world deployment.

Implications for evaluation design are practical: benchmark developers should declare the task class and intended user; retain reference accuracy for stable closed-form tasks; permit multiple acceptable outputs or rubric-based scoring for open-ended tasks; apply evidence grounding and safety gates; and report evaluator qualifications, calibration procedures, and interrater reliability.

## Supplementary material

10.2196/99019 Multimedia Appendix 1 Summary of medical benchmark and evaluation design dimensions.

## Abbreviations

EGFR: epidermal growth factor receptor
LLM: large language model
TAPUR: Targeted Agent and Profiling Utilization Registry
