# Supplementary material for: Challenges of Single-Reference Benchmarks for Clinical Reasoning in Large Language Model–Based Decision Support
Source: J Med Internet Res. 2026 Nov 13;28:e99019. doi: 10.2196/99019 (PMC13766656; doi:10.2196/99019)
Supplement: Supplementary file 1 [file jmir-v28-e99019-s001.docx]

| **Year** | **Dataset Name** | **Evaluation Type** | **Language** | **Dataset Size** | **Question Source** | **Validation Method** | **Validation Category** | **Original Paper Title** | **Evaluation-design dimensions** | | | | | | |
| --- | --- | --- | --- | --- | --- | --- | --- | --- | --- | --- | --- | --- | --- | --- | --- |
|  |  |  |  |  |  |  |  |  | **Reference-output structure** | **Multiple acceptable outputs permitted?** | **Reasoning process explicitly assessed?** | **Uncertainty explicitly assessed?** | **Patient preferences explicitly assessed?** | **Safety explicitly assessed?** | **Guideline concordance explicitly assessed?** |
| **2017** | LiveQA | Knowledge-based | English | 634 medical QA pairs | U.S. National Library of Medicine (NLM) consumer health questions | Medical librarian annotation | Expert annotation | Overview of the Medical Question Answering Task at TREC 2017 LiveQA | Open-ended answer with NLM reference material and human assessment | **Yes** | No | No | No | No | No |
| **2019** | MedicationQA | Knowledge-based | English | 674 medical QA pairs | Real consumer medication questions | Medical expert (including clinicians) verification | Expert annotation | Bridging the Gap Between Consumers’ Medication Questions and Trusted Answers | One curated correct and complete answer from a trusted medication source | No | No | No | No | No | No |
| **2019** | HEAD-QA | Knowledge-based | Spanish | 6,765 questions (including 1,149 medical questions) | Spanish medical licensing examination | Official exam validation | Exam answers | HEAD-QA: A Healthcare Dataset for Complex Reasoning | Single keyed multiple-choice answer | No | No | No | No | No | No |
| **2019** | PubMedQA | Hybrid | English | 211,300 human-generated QA pairs | PubMed abstracts | Expert annotation | Expert annotation | PubMedQA: A Dataset for Biomedical Research Question Answering | One yes/no/maybe label, with a reference long answer in the dataset | No | No | **Yes** | No | No | No |
| **2020** | MedDialog | Practice-based | English / Chinese | 3.4M Chinese dialogues, 260k English dialogues | Online medical consultations | Basic data cleaning, no clinical validation | Real-world data | MedDialog: Large-scale Medical Dialogue Datasets | Recorded doctor response; human rubric used on a downstream dialogue subset | Partial | No | No | No | No | No |
| **2021** | MMLU-Medical | Knowledge-based | English | 1,785 medical questions | Academic or professional examinations | Academic validation, no clinical review | Exam answers | Measuring Massive Multitask Language Understanding | Single keyed multiple-choice answer | No | No | **Yes** | No | No | No |
| **2022** | MedDG | Practice-based | Chinese | 17,864 Chinese dialogues | Online medical consultation platforms | Domain expert annotation | Expert annotation | MedDG: An Entity-Centric Medical Dialogue Dataset | Reference dialogue response plus sampled human ratings | **Yes** | No | No | No | No | No |
| **2021** | MedQA | Knowledge-based | English / Chinese | 61,097 medical and clinical questions | USMLE / Chinese Medical Licensing Exam / Taiwan Medical Exam | MD-level expert verification | Expert annotation | What Disease Does This Patient Have? A Large-scale Open Domain QA Dataset from Medical Exams | Single best-answer multiple-choice key | No | No | No | No | No | No |
| **2022** | ReMeDi | Practice-based | Multilingual | 96,965 dialogues | Multi-domain medical dialogues | Fine-grained medical labeling | Expert annotation | ReMeDi: Resources for Multi-domain Medical Dialogues | Reference dialogue response plus sampled human ratings | **Yes** | No | No | No | No | No |
| **2022** | MedMCQA | Knowledge-based | English | 194,000+ questions | AIIMS / NEET PG medical exams | Compilation of real exam questions | Exam answers | MedMCQA: A Large-scale Multi-subject Medical Multiple Choice QA Dataset | Single keyed multiple-choice answer | No | No | No | No | No | No |
| **2023** | CMExam | Knowledge-based | Chinese | 68,119 questions | Chinese National Medical Licensing Examination | GPT-4 assisted human verification | Exam answers | CMExam: A Comprehensive Chinese Medical Examination Benchmark | Keyed option or option set plus one reference solution explanation | No | **Yes** | No | No | No | No |
| **2023** | HealthSearchQA | Knowledge-based | English | 3,173 high-frequency medical search questions | Search engine queries | Consumer validation | Real-world data | Large Language Models Encode Clinical Knowledge | Open-ended long-form answer with clinician reference and multidimensional rubric | **Yes** | **Yes** | No | No | **Yes** | Partial |
| **2024** | MedBench-Edu | Knowledge-based | Chinese | 40,041 questions | Chinese medical licensing, residency, attending physician exams, and real cases | Four-stage medical exam validation | Exam answers | MedBench: A Large-scale Chinese Medical Benchmark | Mixed keyed examinations and open clinical-case outputs | Partial | Partial | No | No | No | No |
| **2025** | JAMA Clinical Challenge & Medbullets | Practice-based | English | 308 USMLE-style questions + 1,524 JAMA cases | USMLE Step 2/3 and JAMA | Expert-written explanations | Expert annotation | Benchmarking Large Language Models on Challenging Medical Questions | Single keyed option plus expert-written explanation | **Yes** | **Yes** | No | No | No | No |
| **2024** | MedExQA | Knowledge-based | English | 965 questions | Five medical specialties | Multi-expert explanations | Expert annotation | MedExQA: A Medical Exam Question Answering Benchmark | Single keyed option plus two expert explanations per item | **Yes** | **Yes** | No | No | No | No |
| **2024** | MedBench-Platform | Knowledge-based | Chinese | 300,901 questions | Chinese medical examinations | Standardized cloud-based evaluation | Exam answers | MedBench-Platform: A Unified Medical Benchmarking System | Mixed keyed tasks and open outputs across five capability dimensions | Partial | Partial | No | No | **Yes** | No |
| **2024** | MedQA-SWE | Practice-based | Swedish | 3,180 questions | Swedish medical licensing examination | Manual curation | Exam answers | MedQA-SWE: A Swedish Medical Question Answering Dataset | Single keyed multiple-choice answer | No | No | No | No | No | No |
| **2024** | KorMedMCQA | Knowledge-based | Korean | 7,469 questions | Korean medical licensing examination | Chain-of-thought reasoning evaluation | Exam answers | KorMedMCQA: Korean Medical Multiple Choice QA Dataset | Single keyed multiple-choice answer | No | Partial | No | No | No | No |
| **2024** | BiasMedQA | Knowledge-based | English | 1,273 questions | MedQA with bias-inducing prompts | Cognitive bias validation | Exam answers | BiasMedQA: Evaluating Cognitive Biases in Medical QA | Single keyed multiple-choice answer under bias-inducing prompts | No | No | No | No | No | No |
| **2024** | DiversityMedQA | Knowledge-based | English | 2,109 questions | MedQA with demographic perturbations | GPT-4 filtered validation | Exam answers | DiversityMedQA: Evaluating Demographic Bias in Medical QA | Single keyed answer under demographic perturbations | No | No | No | No | No | No |
| **2024** | ClinicBench | Practice-based | English | 17 datasets, 11 tasks | Clinical scenarios | Medical expert evaluation | Expert annotation | ClinicBench: Benchmarking Clinical Reasoning of LLMs | Mixed structured targets and open-ended clinical outputs | Partial | Partial | No | No | Partial | No |
| **2024** | CliMedBench | Practice-based | Chinese | 33,735 questions | Real-world medical reports | Expert-guided construction | Expert annotation | CliMedBench: A Chinese Clinical Medical Benchmark | Open and structured outputs across 14 clinical scenarios | **Yes** | Partial | No | No | **Yes** | Partial |
| **2024** | MedQA-CS | Practice-based | English | 1,667 instruction samples | OSCE-inspired scenarios | AI-SCE framework | AI-generated | MedQA-CS: An OSCE-style Clinical Scenario QA Benchmark | OSCE-style free-text and role-play responses scored by AI-SCE criteria | **Yes** | **Yes** | No | No | No | No |
| **2024** | TCMBench | Knowledge-based | Chinese | 5,473 questions | Traditional Chinese Medicine licensing examination | Official exam scoring | Exam answers | TCMBench: Benchmarking Large Language Models on Traditional Chinese Medicine | Keyed answer plus generated analysis or explanation | **Yes** | **Yes** | No | No | No | No |
| **2025** | DentalBench | Knowledge-based | Bilingual | 36,597 questions | 16 dental subdomains | Expert curation | Expert annotation | DentalBench: A Comprehensive Dental Question Answering Benchmark | Mixed single-answer, multi-answer, open-ended, and definition tasks | Partial | No | No | No | No | No |
| **2025** | LLMEval-Med | Practice-based | Multilingual | 2,996 questions | Real EHRs + expert-designed scenarios | Physician verification | Expert annotation | LLMEval-Med: Evaluating Large Language Models in Medicine | Open-ended responses scored with expert-developed checklists | **Yes** | **Yes** | No | No | **Yes** | No |
| **2025** | AfriMed-QA | Knowledge-based | English | 15,275 questions | Pan-African medical colleges | 621 contributors | Expert annotation | AfriMed-QA: A Pan-African Medical Question Answering Dataset | Mixed closed questions and open short-answer or consumer-question responses | Partial | Partial | No | No | Partial | Partial |
| **2024** | MVME | Practice-based | Chinese | 506 high-quality cases | Chinese clinical case websites | Multi-agent simulation | Real-world data | Multi-View Medical Evaluation Benchmark | Multi-turn clinical report scored across five report components | **Yes** | **Yes** | No | No | No | No |
| **2024** | ChatCoach | Practice-based | English | 3,500 dialogue rounds (13,666 utterances) | Real medical consultations + synthetic coaching | Medical staff / students + GPT-4 | Physician annotation | ChatCoach: Benchmarking LLMs on Medical Communication Coaching | Generated coaching feedback with human quality ratings | **Yes** | No | No | No | No | No |
| **2025** | MEDEC | Practice-based | English | 3,848 clinical texts | U.S. hospital systems | Medical annotator verification | Physician annotation | MEDEC: Medical Dataset for Evaluation of Clinical Language Models | Error flag or sentence plus corrected clinical text | **Yes** | No | No | No | No | No |
| **2025** | MedXpertQA | Knowledge-based | English | 4,460 questions (including multimodal) | USMLE, COMLEX-USA, specialty exams, medical imaging | Multi-round licensed physician verification | Physician annotation | MedXpertQA: A Multimodal Medical Expert-Level QA Benchmark | Single keyed multiple-choice answer with a reasoning-focused subset | No | No | No | No | No | No |
| **2025** | TRINDs | Knowledge-based | Multilingual | 11,719 prompts | Authoritative medical resources | Quality evaluation by 7 experts | Expert annotation | TRINDs: Trusted Instruction Dataset for Medical LLMs | Reference diagnosis with semantic-equivalence autorating | **Yes** | No | No | No | No | No |
| **2025** | HealthBench | Practice-based | Multilingual | 5,000 multi-turn dialogues | Physician-designed scenarios | 262 physicians (60 countries) | Expert annotation | HealthBench: Evaluating Large Language Models Toward Improved Human Health | Conversation-specific physician rubric with no single answer key | **Yes** | No | **Yes** | No | **Yes** | No |
| **2025** | MedAgentBench | Practice-based | English | 300 patient-level tasks | Stanford hospital records | Licensed physician curation | Physician annotation | MedAgentBench: A Virtual EHR Benchmark for Medical Agents | Task-success criterion based on returned answer or target EHR state | **Yes** | No | No | No | No | No |
| **2025** | CSEDB | Practice-based | English | 2,069 open-ended questions | 26 clinical departments, 30 evaluation metrics | 32 specialist physicians | Physician annotation | CSEDB: Clinical Safety and Effectiveness Diagnostic Benchmark | Open response plus expert binary or graded scoring rules | **Yes** | No | No | No | **Yes** | **Yes** |
| **2025** | MedHELM | Hybrid | English | 35 benchmarks (17 existing + 18 new) | 121 clinical tasks | Validation by 29 clinicians | Physician annotation | MedHELM: A Holistic Evaluation of Medical LLMs | Mixed exact-match targets and task-specific rubrics across 35 benchmarks | Partial | No | Partial | No | Partial | Partial |
| **2025** | MENTAT | Practice-based | English | 203 questions | Mental health scenarios | Clinical expert annotation | Physician annotation | MENTAT: Benchmarking LLMs in Mental Health Scenarios | Five options with expert validity ratings and preference distributions for ambiguous tasks | **Yes** | No | Partial | No | No | No |
| **2025** | EBMQA | Hybrid | English | 105,222 questions | 50k papers + 20M medical relations | Medical expert validation | Expert annotation | EBMQA: Evidence-Based Medical Question Answering Benchmark | Multiple-choice: one correct numeric answer or up to five correct semantic answers, plus IDK | **Yes** | No | **Yes** | No | No | No |
| **2025** | DiagnosisArena | Practice-based | English | 1,113 cases | 10 top-tier medical journals | Expert screening and review | Expert annotation | DiagnosisArena: Benchmarking Diagnostic Reasoning of LLMs | One reference diagnosis judged for semantic identity; top-k diagnosis list | **Yes** | No | No | No | No | No |
